# Surgical decision-making in superior canal dehiscence syndrome with concomitant otosclerosis

**DOI:** 10.1007/s00405-024-08679-w

**Published:** 2024-05-23

**Authors:** S. W. Van Dijk, J. P. M. Peters, R. J. Stokroos, H. G. X. M. Thomeer

**Affiliations:** Department of Otorhinolaryngology and Head and Neck Surgery, University Medical Center Utrecht, Heidelberglaan 100, 3584 CX Utrecht, Netherlands

**Keywords:** Superior canal dehiscence syndrome, Otosclerosis, Capping, Plugging, Resurfacing, Stapedotomy

## Abstract

**Objective:**

The diagnosis and management of Superior Canal Dehiscence Syndrome (SCDS) with concomitant otosclerosis can be a challenge. Otosclerosis can mask SCDS symptoms and stapes surgery may reveal or exacerbate vestibular symptoms. Our aim is to present four cases of SCDS with concomitant otosclerosis and thereby informing the reader about the possibility of this dual occurrence and its implications for treatment.

**Cases:**

Four patients with SCDS and concomitant otosclerosis are presented. Two patients underwent surgical treatment for both SCDS and otosclerosis and two patients opted for conservative management.

**Outcomes:**

The main differences between surgically and non-surgically treated cases are the presence of autophony and pressure-induced vertigo and a more severe experience of symptoms in surgically treated cases. Surgically treated cases achieved a sizeable reduction in postoperative air–bone gap and resolution of vestibular symptoms.

**Conclusion:**

The subjective severity of symptoms in combination with shared decision-making is key in determining the appropriate treatment plan for SCDS and concomitant otosclerosis.

## Introduction

Superior semicircular canal dehiscence syndrome (SCDS) is an otovestibular condition involving a dehiscence of the superior semicircular canal [1]. It can cause a variety of audiological and vestibular symptoms, including hearing loss, autophony, pulsatile tinnitus, vertigo and hyperacusis [2–4]. Possible clinical signs include Tullio’s sign and Hennebert’s sign, which describe the occurrence of vertigo or nystagmus after exposure to loud sounds or pressure, respectively [2, 3, 5–7]. The superior semicircular canal dehiscence (SCD) is detected using a high-resolution (HR) CT-scan of the mastoid, with reconstructions in the Pöschl plane [8, 9]. The diagnosis of SCDS also requires appropriate symptomatology and instrumental findings (e.g. VEMP, video head impulse test (vHIT)) [10–15]. There is a positive correlation between the size of SCD and air-conduction (AC) thresholds and air–bone gap (ABG) [3, 16–19]. A location closer to the ampulla or a larger dehiscence is associated with a lower cervical vestibular-evoked myogenic potential (VEMP) threshold and presence of auditory symptoms [3, 16, 19–21]. Surgical treatment is only indicated in a minority of patients and may consist of resurfacing or plugging the dehiscence via a middle cranial fossa approach (MCFA) or transmastoid approach (TMA) [1, 22].

The distinction between SCD and SCDS is an important one. In the latter case, the SCD is associated with appropriate symptoms which may require treatment. Current literature frequently reports the presence of SCD, which may be asymptomatic, and therefore clinically irrelevant [23–25]. Using these cases as a basis for the management of SCDS and otosclerosis may understate the risk of exacerbation or unmasking of vestibular symptoms after stapedotomy [26, 27]. This is further complicated by a relatively large variation in symptomatology and degrees of SCDS [28, 29].

Otosclerosis is an osseous dysplasia of the otic capsule in the temporal bone which predominantly leads to hearing loss [30, 31]. It typically presents with progressive conductive hearing loss without evidence for middle ear inflammation [30]. It can be detected using a CT-scan of the middle ear [32, 33], however, the additional value of CT is limited [34]. The diagnosis can be confirmed by testing the mobility of the stapes during middle ear inspection. Treatment may consist of stapedotomy or hearing aid(s) [35].

SCDS can mimic otosclerotic symptoms and stapes fixation can eliminate the third window effect and thereby mask SCDS symptoms [36]. In these patients, stapes surgery can reveal or exacerbate SCDS-related symptoms (e.g. sound/pressure induced vertigo, hyperacusis, autophony, pulsatile tinnitus and conductive/mixed hearing loss) [23–25, 37–40]. In patients with otosclerosis, AC VEMP may be less reliable than bone-conduction (BC) VEMP due to impedance of sounds transmission through the ossicular chain [41, 42].

Furthermore, in patients with SCDS and concomitant otosclerosis, the success rate of stapes surgery in reducing the ABG may be impaired, as a postoperative ABG of less than 10 dB SPL is only achieved in 60% of cases [23, 24]. In case of failed stapes surgery, the presence of SCD should be investigated as a possible cause [24, 25, 29, 39, 40, 43–46].

Appropriate indications for and a comparison of surgical treatment options for SCDS with concomitant otosclerosis cannot be determined due to the limited number of cases and heterogeneous reported outcome measures [22, 38].

The goal of this case series is to review four patient cases regarding the presentation and treatment of SCDS with concomitant otosclerosis, and thereby informing the reader about the possibility of this dual occurrence and its implications with regards to treatment.

## Case one

A 42-year-old woman presented with a history of aural fullness of the left ear since two years, autophony, asymmetric hearing loss and vertigo following loud noises. She underwent ossicular chain reconstruction of the right ear prior to the occurrence of the aforementioned complaints. Otoscopic examination of the left ear showed no abnormalities. Hennebert’s sign was positive. Tone audiometry (see Table 1) showed supranormal BC and subnormal low-frequency AC thresholds in the left ear. Stapes reflexes could not be evoked ipsi- and contralaterally. BC ocular VEMP (oVEMP) revealed a lowered threshold of 95 dB SPL in both ears. CT imaging of the mastoid bone confirmed a short interruption of the bony coverage of the left SSC (see Fig. 1.1). The dehiscence was plugged using bone wax via MCFA. After surgery, the autophony and vertigo resolved and the BC oVEMP thresholds were normalized. The BC threshold normalised, but AC hearing loss persisted. Stapes reflexes could still not be evoked in the left ear. In a second surgery, otosclerosis was confirmed and a stapedotomy was performed in the left ear. Postoperative audiometry revealed a sizeable reduction of the ABG. The only remaining complaint after the two surgeries was persistent tinnitus in the left ear.

**Table 1:** Tone audiometry results of all cases pre- and postoperatively and comparison to current literature

	Pre-operative			Postoperative			Difference in ABG
	BC	AC	ABG	BC	AC	ABG	
Case one*	− 12	42	54	8	18	10	− 44
Case two	10	52	42	23	40	17	− 25
Case three	0	20	20	NA	NA	NA	NA
Case four	18	47	29	NA	NA	NA	NA
Yong et al. [36]	22	70	48	NA	NA	NA	NA
	2	50	48	NA	NA	NA	NA
	12	47	35	NA	NA	NA	NA
Dewyer et al. [25]	23	65	42	15	40	25	− 17
	25	80	55	17	52	35	− 20
	8	65	57	NA	NA	NA	NA
	7	45	38	7	47	40	+ 2
	− 2	35	37	− 8	23	31	− 6
	17	45	28	13	32	19	− 9
	7	27	20	NA	NA	NA	NA
	12	30	18	8	40	32	+ 14
	50	88	38	NA	23	NA	NA
Maxwell et al. [39]	NA	77	NA	NA	55	NA	NA
	NA	47	NA	NA	62	NA	NA
	18	55	37	NA	NA	NA	NA
	NA	97	NA	NA	53	NA	NA
	NA	97	NA	NA	65	NA	NA
Pritchett et al. [47]	17	53	36	13	23	10	− 26
	23	67	44	12	23	11	− 33

All tone audiometry results pertain to the most affected ear and are presented as the average dB SPL of thresholds at 250, 500 and 1000 Hz

*Comparison of pre-operative results with results after both SCD repair and stapedotomy

*AC*  air conduction, *BC*  bone conduction, *ABG*  air–bone gap, *NA* not available

**Fig. 1:**
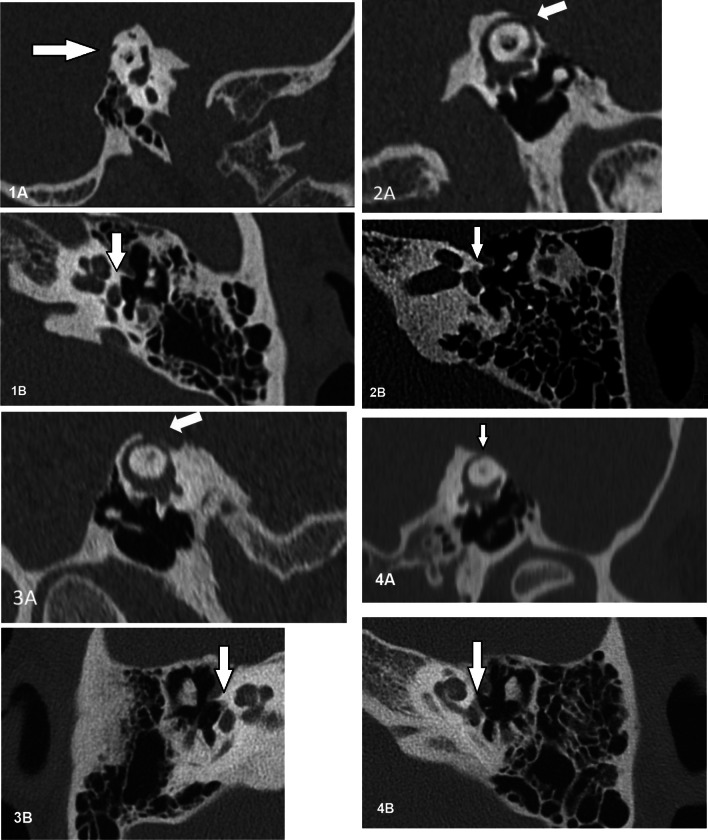
Computed tomography imaging of the mastoid bone in all patients. **1A** Case one, Pöschl view showing a dehiscence of the left superior semicircular canal. **1B** Case one, axial view of the left temporal bone showing the fissula ante fenestram. **2A** Case two, Pöschl view showing a dehiscence of the left superior semicircular canal. **2B** Case two, axial view of the left temporal bone showing fenestral otosclerosis. **3A** Case three, Pöschl view showing a dehiscence of the right superior semicircular canal. **3B** Case three, axial view of the right temporal bone revealing fenestral otosclerosis. **4A** Case four, Pöschl view showing a dehiscence of the left superior semicircular canal. **4B** Case four, axial view of the left temporal bone showing fenestral otosclerosis

## Case two

A 58-year-old woman presented with a history of hyperacusis, autophony, and hearing loss of the left ear, and an unstable sensation when walking. Tullio’s and Hennebert’s signs were both positive. Otoscopic examination revealed no abnormalities. Tone audiometry revealed increased AC thresholds in the left ear (see Table 1) and normal hearing in the right ear. CT imaging of the mastoid revealed a bilateral dehiscence of the SCCs (see Fig. 1.2). BC oVEMP and electronystagmography (ENG) revealed no abnormalities. The patient underwent middle ear inspection revealing a fixed stapes, which confirmed otosclerosis in the left ear. First, a stapedotomy was performed, and subsequently the left SSC was covered via TMA using auricular cartilage, during the same surgery. After surgery, the patient reported resolution of vertiginous symptoms and improved hearing in the left ear. Tone audiometry revealed a reduced ABG (see Table 1).

## Case three

A 28-year-old woman presented with a history of pressure sensation in the right ear since one year, hyperacusis, and pulsatile tinnitus (not synchronous to the heart rate). Otoscopic examination and audiometry (see Table 1) revealed no abnormalities. Oscillopsia was present and Tullio’s sign was positive. CT imaging confirmed a dehiscence of the right SSC and ipsilateral fenestral otosclerosis (see Fig. 1.3). BC oVEMP revealed lowered thresholds and increased amplitudes in the right ear. ENG showed no abnormalities. The patient preferred not to undergo surgery due to the relatively limited and mild symptoms.

## Case four

A 25-year-old man presented with a history of progressive hearing loss mainly affecting the left ear, tinnitus, hyperacusis and the sensation of pressure in the left ear. He previously underwent grommet placement and stapedotomy of the left ear. Otoscopic examination showed a grommet in situ in the left ear and no abnormalities in the right ear. Tullio's sign was positive. Tone audiometry revealed a bilateral mixed hearing loss (see Table 1). CT imaging of the mastoid showed a dehiscence of the left SCC in combination with bilateral otosclerosis (see Fig. 1.4). BC oVEMP revealed an asymmetric response threshold of 120 dB SPL in the right ear and 110 dB SPL in the left ear. The patient opted not to have surgery due to the relatively mild symptoms.

## Discussion

This study was conducted at the University Medical Centre (UMC) Utrecht The Netherlands, in compliance with the principles of the Declaration of Helsinki. This study was exempt from approval of an ethics committee under Dutch law.

### Case discussion

The strength of our approach in these cases was appropriate use of shared decision making, thereby omitting surgery in two cases. A limitation is not combining the treatment of otosclerosis in the first case along with SCD repair. This would have made the treatment for both conditions possible in one operation.

The only discernible difference in complaints between patients who did and did not receive surgical treatment is the presence of autophony and pressure-induced vertigo in surgically treated cases (see Table 2). The tipping point in opting for surgical treatment apparently lies in the subjective severity of symptoms, which underlines the importance of shared decision making.

**Table 2:** Patient characteristics at presentation

Symptom	Surgically treated cases		Conservatively managed cases	
	Case one	Case two	Case three	Case four
Pulsatile tinnitus	+	+	+	+
Sound-induced nystagmus or vertigo	+	+	+	+
Pressure-induced nystagmus or vertigo	+	+	−	−
Autophony	+	+	−	−
Hyperacusis	+	+	+	+
Brain fog	−	+	−	−
Hearing loss	+	+	+	−
Aural fullness	+	+	+	+
Balance disorder	−	+	−	+
Oscillopsia	−	−	−	+

### Overview of literature

In our surgical cases, a larger reduction in ABG was achieved (− 44 dB SPL and − 25 dB SPL) compared to the average reduction reported in literature (− 14 dB SPL; see Table 1). One possible explanation for this difference could be that almost all literature reports the presence of SCD, not of SCDS. It could be that SCDS is accompanied by worse hearing loss compared to SCD and therefore facilitates a larger improvement after surgical intervention.

Current literature fails to reveal evidence-based prognostic factors for the outcome of stapedotomy in patients with SCDS and comorbid otosclerosis. Caution is therefore advised when considering treating otosclerosis with stapedotomy in these patients, as it may not achieve the desired result and may even unmask vestibular symptoms [23–25, 37–40]. However, stapedotomy is not necessarily contra-indicated in these cases. It can lead to adequate closure of the ABG without unmasking vertiginous symptoms [39, 44, 45, 47] (Table 3).

**Table 3:** Summary of test results of all cases at presentation

Test	Case one	Case two	Case three	Case four
Tone audiometry	Conductive hearing loss AS	Conductive hearing loss AS	Mixed hearing loss ADS	No hearing loss
Stapes reflex	Negative ADS	Not tested	Not tested	Not tested
Ocular vestibular evoked myogenic potential	Asymmetric lowered threshold	No abnormalities	Asymmetric lowered threshold	Asymmetric lowered threshold
Electronystagmography	Not tested	No abnormalities	Not tested	No abnormalities

**Fig. 2:**
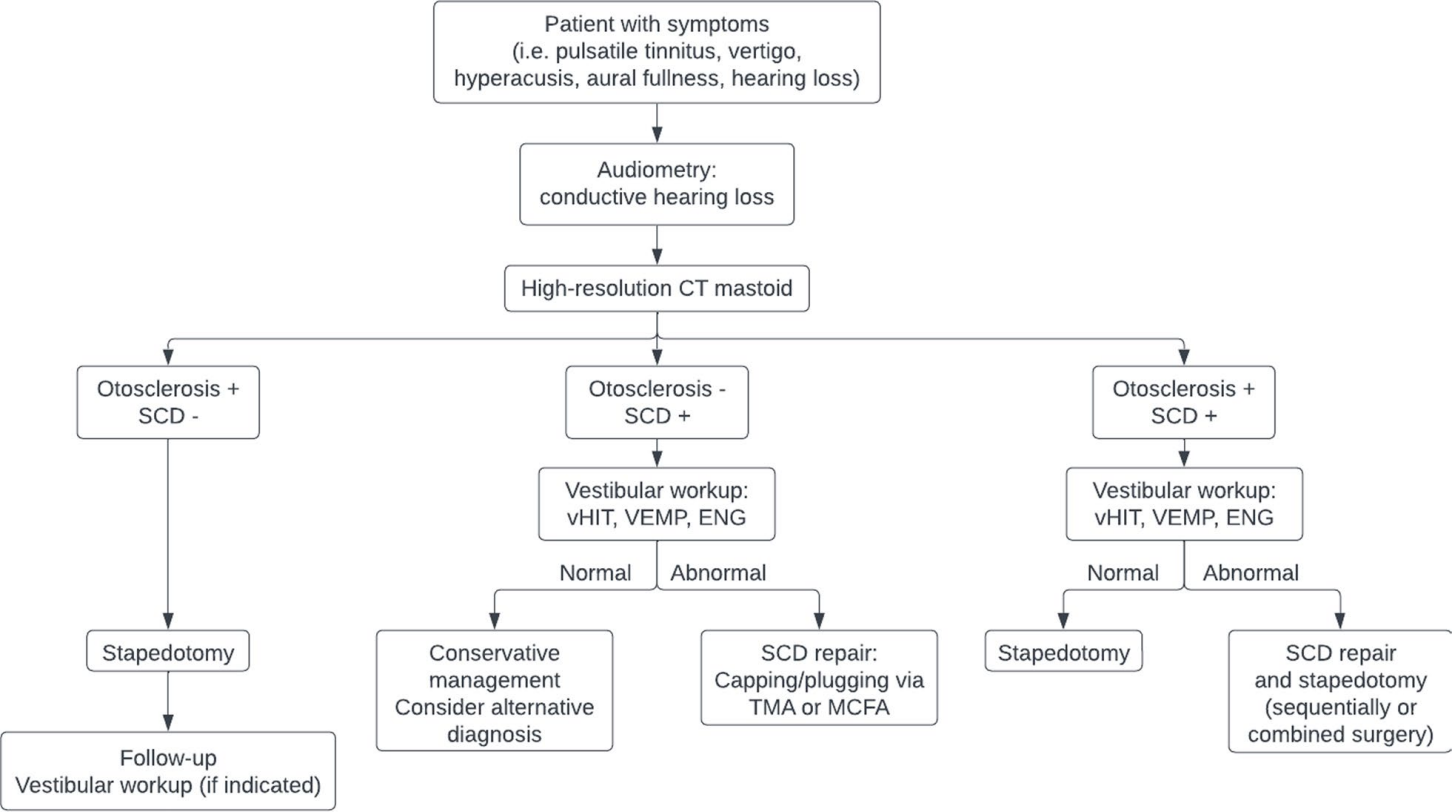
Strategic decision-making flowchart. Flowchart depicting the recommended decision-making process for patients with otosclerosis and/or SCD. In case of isolated otosclerosis, a stapedotomy may be indicated. In case of isolated SCD, shared decision-making should guide you towards either conservative management or surgical SCD repair. In case of both otosclerosis and SCD, surgical management via stapedotomy and/or SCD repair may be indicated. It is important to underline that shared decision-making should play a key role in this process

We created a strategic decision-making flowchart (see Fig. 2) based on current literature, our cases and previous discussion points. This flowchart displays a compact overview of the suggested diagnostic and decision-making process, which may prove beneficial for clinicians in the future. The presence of concomitant SCDS should be considered and investigated using HRCT of the mastoid in cases of atypical otosclerosis (i.e. otosclerosis does not explain all present symptoms). In cases with SCD, a vestibular workup (consisting of vHIT, BC VEMP and ENG) is recommended. This can aid in determining whether the SCD may be a likely cause of the patients' complaints and may therefore lead to different treatment strategies. If the vestibular workup is normal, a more conservative approach may be appropriate.

## Conclusion

It remains unclear from literature and our presented cases what the best treatment strategy is in cases with both otosclerosis and SCDS. The decision-making process in terms of management strategies should be principally influenced by the subjective severity of symptoms experienced by the patient.

## Data Availability

Study data are readily available for the coming 15 years. These can be provided on request at the corresponding author.
